# Evaluation of synaptotagmin‐1 action models by all‐atom molecular dynamics simulations

**DOI:** 10.1002/2211-5463.13966

**Published:** 2025-01-15

**Authors:** Josep Rizo, Klaudia Jaczynska, Christian Rosenmund

**Affiliations:** ^1^ Department of Biophysics University of Texas Southwestern Medical Center Dallas TX USA; ^2^ Department of Biochemistry University of Texas Southwestern Medical Center Dallas TX USA; ^3^ Department of Pharmacology University of Texas Southwestern Medical Center Dallas TX USA; ^4^ Institute of Neurophysiology Charité – Universitätsmedizin Berlin, Corporate Member of Freie Universität Berlin and Humboldt‐Universität zu Berlin Germany; ^5^ NeuroCure Cluster of Excellence Berlin Germany

**Keywords:** membrane fusion, molecular dynamics simulation, neurotransmitter release, SNAREs, synaptic vesicle fusion, synaptotagmin

## Abstract

Neurotransmitter release is triggered in microseconds by the two C_2_ domains of the Ca^2+^ sensor synaptotagmin‐1 and by SNARE complexes, which form four‐helix bundles that bridge the vesicle and plasma membranes. The synaptotagmin‐1 C_2_B domain binds to the SNARE complex via a ‘primary interface’, but the mechanism that couples Ca^2+^‐sensing to membrane fusion is unknown. Widespread models postulate that the synaptotagmin‐1 Ca^2+^‐binding loops accelerate membrane fusion by inducing membrane curvature, perturbing lipid bilayers or helping bridge the membranes, but these models do not seem compatible with SNARE binding through the primary interface, which orients the Ca^2+^‐binding loops away from the fusion site. To test these models, we performed molecular dynamics simulations of SNARE complexes bridging a vesicle and a flat bilayer, including the synaptotagmin‐1 C_2_ domains in various configurations. Our data do not support the notion that insertion of the synaptotagmin‐1 Ca^2+^‐binding loops causes substantial membrane curvature or major perturbations of the lipid bilayers that could facilitate membrane fusion. We observed membrane bridging by the synaptotagmin‐1 C_2_ domains, but such bridging or the presence of the C_2_ domains near the site of fusion hindered the action of the SNAREs in bringing the membranes together. These results argue against models predicting that synaptotagmin‐1 triggers neurotransmitter release by inducing membrane curvature, perturbing bilayers or bridging membranes. Instead, our data support the hypothesis that binding via the primary interface keeps the synaptotagmin‐1 C_2_ domains away from the site of fusion, orienting them such that they trigger release through a remote action.

## Graphical abstract

We used molecular dynamics simulations to test models proposing that synaptotagmin‐1 facilitates membrane fusion by perturbing lipid bilayers, inducing membrane curvature and/or bridging membranes. The simulations show that synaptotagmin‐1 C_2_‐domains placed near the site of fusion hinder the ability of SNARE complexes to bring the membranes together and induce fusion, supporting alternative models whereby synaptotagmin‐1 acts remotely to accelerate fusion.

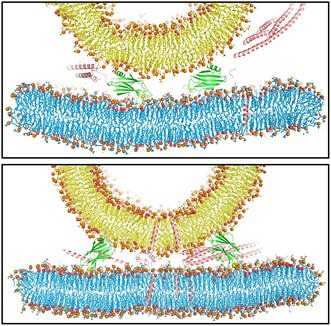

**Abbreviations**
Jxt: juxtamembrane
MD: molecular dynamics
NMR: nuclear magnetic resonance
NSF: *N*‐ethylmaleimide sensitive factor
PIP_2_
: phosphatidylinositol 4,5‐bisphosphate
SNAP: soluble NSF attachment protein
SNAP‐25: synaptosomal associated protein 25 kDa
SNARE: SNAP receptor
Syt1: synaptotagmin‐1
TM: transmembrane

Communication between neurons is mediated by neurotransmitters that are released by Ca^2+^‐evoked synaptic vesicle exocytosis. This process requires tethering of synaptic vesicles to presynaptic active zones and priming to a release‐ready state(s) that enables very fast vesicle fusion upon Ca^2+^ influx into a presynaptic terminal [1] (< 60 μs in fast synapses [2]). Extensive studies have allowed reconstitution of the basic steps that lead to exocytosis with the main components of the neurotransmitter release machinery [3, 4, 5, 6] and definition of their functions [7, 8, 9]. The SNAP receptors (SNAREs) synaptobrevin, syntaxin‐1, and SNAP‐25 mediate membrane fusion [10] by forming four‐helix bundles (SNARE complexes) that bring membranes together as they assemble (‘zipper’) from the N‐ to the C terminus [11, 12, 13, 14], likely catalyzing lipid acyl chain encounters at the polar bilayer‐bilayer interface [15]. These complexes are disassembled after fusion by *N*‐ethylmaleimide sensitive factor (NSF) and soluble NSF attachment proteins (SNAPs) [11], and their assembly is orchestrated by Munc18‐1 and Munc18‐1 [3, 16] via an NSF‐SNAP‐resistant mechanism that starts with Munc18‐1 bound to a closed conformation of syntaxin‐1 [17, 18]. Munc18‐1 later binds also to synaptobrevin and thus forms a template for SNARE assembly [19, 20, 21, 22] while Munc13 bridges the vesicle and plasma membranes [4, 23], and opens syntaxin‐1 [24, 25]. The resulting partially assembled SNARE complexes bind to Synaptotagmin‐1 (Syt1) [26] and to complexin [27], forming a primed macromolecular assembly [28] that hinders fusion but is ready to trigger fast vesicle fusion when Ca^2+^ binds to Syt1 [29].

Despite these advances, it has been difficult to elucidate how Syt1 triggers release. This synaptic vesicle protein contains two C_2_ domains (C_2_A and C_2_B) that form most of its cytoplasmic region and bind three and two Ca^2+^ ions, respectively, via loops at the tip of β‐sandwich structures [30, 31, 32]. Release is triggered by Ca^2+^‐dependent phospholipid binding to these loops [29, 33] and depends more critically on Ca^2+^‐binding to the C_2_B domain than on Ca^2+^‐binding to C_2_A [34, 35]. This predominant role most likely arises because the C_2_B domain binds to phosphatidylinositol 4,5‐bisphosphate (PIP_2_) through a polybasic region on one side of the β‐sandwich [36] and to the SNARE complex through a so‐called primary interface on the other side [26], which sets up the primed state that is ready for fast release [28]. Overwhelming evidence supports the physiological relevance of Syt1 binding to the SNARE complex via the primary interface [26, 37, 38, 39, 40, 41] but at the same time raises a conundrum because such binding orients the Ca^2+^‐binding loops of the Syt1 C_2_B domain away from the site of fusion. In contrast, widely accepted models predict that Syt1 facilitates fast membrane fusion because Ca^2+^‐induced insertion of these loops into membranes [42, 43] perturbs the bilayers [42, 44, 45] and/or induces membrane curvature [46, 47]. Another popular model proposed that Syt1 facilitates fusion by bridging the two membranes [48, 49], helping the SNAREs to overcome the repulsion between the bilayers. However, molecular dynamics (MD) simulations [15, 28] and the extended bilayer‐bilayer interfaces observed in SNARE‐mediated liposome fusion reactions [50] suggest that the SNAREs alone readily bring membranes into contact.

Here we present MD simulations that were designed to investigate the merits of various models of how Syt1 might accelerate membrane fusion and thus shed light into how Syt1 triggers neurotransmitter release. Our results suggest that, when placed close to the site of fusion, the Syt1 C_2_ domains hinder the ability of the neuronal SNARE complex to bring the membranes together and induce fusion. Moreover, the Syt1 C_2_ domains did not cause strong bilayer perturbations or membrane curvature. Our data provide an explanation for why the primary interface orients the Syt1 C_2_B Ca^2+^‐binding loops away from the site of fusion, suggest that models postulating that Syt1 facilitates membrane fusion directly at the site of fusion need to be abandoned, and favor models predicting a remote action of Syt1 such as the recently proposed lever hypothesis [40, 41]. Our simulations also have general implications to understand the mechanisms of action of a variety C_2_ domain proteins that are involved in membrane traffic and include multiple Syt isoforms [51, 52], suggesting that, similar to Syt1, they function remotely rather than directly at the site of membrane fusion.

## Materials and methods

Molecular dynamics simulations were performed with the same methodology employed in our previous simulations [15, 28], using Gromacs [53, 54] with the CHARMM36 force field [55]. System setup was performed at the BioHPC supercomputing facility of UT Southwestern, whereas all minimizations, equilibration steps, and production MD simulations were carried out on Frontera at the Texas Advanced Computing Center (TACC). pymol (Schrödinger, LLC, 1540 Broadway, 24th floor, New York, NY 10036) was used for system design, manual manipulation, and system visualization. The lipid compositions of the vesicle and the flat bilayers resembled those of synaptic vesicles and plasma membranes, respectively [56, 57], and are listed in Table 1 together with other parameters of the simulations. All systems were solvated with explicit water molecules (TIP3P model), adding potassium and chloride ions to reach a concentration of 145 mm and make the system neutral. All Syt1 C_2_AB molecules had five Ca^2+^ ions placed at the corresponding binding sites [31, 32].

**Table 1:** Parameters of the MD simulations^a^.

	CHL1	POPC	SAGL	SAPE	SAPI2D	SDPE	SDPS	SOPS	Total
Vesicle									
Outer leaflet	1258	296	0	534	0	282	210	199	2779
%	45.3	10.6	0	19.2	0	10.1	7.6	7.2	
Inner leaflet	814	668	0	183	0	99	1	1	1766
%	46.1	37.8	0	10.4	0	5.6	0.1	0.1	
Flat bilayer 1									
Upper leaflet	830	151	18	132	93	262	182	181	1849
%	44.9	8.2	1.0	7.1	5.0	14.2	9.8	9.8	
Lower leaflet	810	774	18	72	0	126	0	0	1800
%	45.0	43.0	1.0	4.0	0	7.0	0	0	
Flat bilayer 2									
Upper leaflet	748	130	17	115	82	231	165	161	1649
%	45.3	7.9	1.0	7.0	5.0	14.0	10.0	9.8	100
Lower leaflet	720	688	16	64	0	112	0	0	1600
%	45.0	43.0	1.0	4.0	0	7.0	0	0	100

System name	Flat bilayer	Number of atoms	Initial cell dimensions	Length and temperature
cac2absc	1	4,921,862	36.9 × 36.4 × 36.7 nm^3^	668 ns, 310 K
fusiong	1	4,922,564	36.9 × 36.4 × 36.7 nm^3^	510 ns, 310 K
nosytfusion	1	4,921,880	36.9 × 36.4 × 36.7 nm^3^	510 ns, 310 K
sytfusion2g	1	4,920,453	36.9 × 36.4 × 36.7 nm^3^	570 ns, 310 K
sytfusion3	2	4,586,690	35.6 × 36.8 × 35.3 nm^3^	450 ns, 310 K

^a^ All systems included four trans‐SNARE complexes, a 24 nm vesicle and the indicated flat bilayer. The cac2absc, fusiong and sytfusion3 systems had four C_2_AB molecules, sytfusion2g had two and nosytfusion had none. C_2_AB molecules had five bound Ca^2+^ ions.

**Fig. 1:**
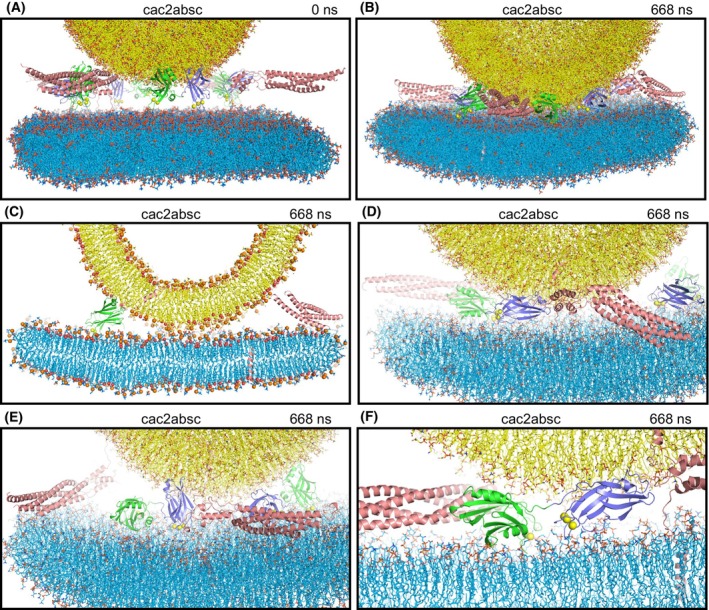
Molecular dynamics simulation designed to investigate whether Ca^2+^‐saturated Syt1 C_2_ domains spontaneously insert into the bilayers, or perturb or bridge the bilayers (cac2absc simulation). (A) Initial configuration of the system. (B–F) Different views of the configuration after 668 ns of simulation showing the overall system (B), a slice of the center of the system to illustrate the contact between the vesicle and the flat bilayer (C), close‐up views showing the SNARE complexes and C_2_AB molecules on one side of the system (D) or the other side (E), and another close‐up view showing a C_2_B domain bound to the C terminus of a SNARE complex that is almost fully assembled and with the Ca^2+^‐binding loops oriented toward the center of the membrane‐membrane interface (F). Lipids are shown as stick models with nitrogen atoms in dark blue, oxygens in red, phosphorus in orange and carbon atoms in yellow (vesicle) or light blue (flat bilayer). Proteins are represented by ribbon diagrams, with SNARE complexes in salmon color, Syt1 C_2_A domain in slate blue and Syt1 C_2_B domain in green. Ca^2+^ ions are shown as yellow spheres. In (C), phosphorous atoms of phospholipids and oxygen atoms of cholesterol molecules are shown as spheres to illustrate the approximate locations of lipid head groups. Note that, because the slice shown is thin, only portions of some of the proteins, if any, are seen. The same color coding was used in all the figures except when noted otherwise.

**Fig. 2:**
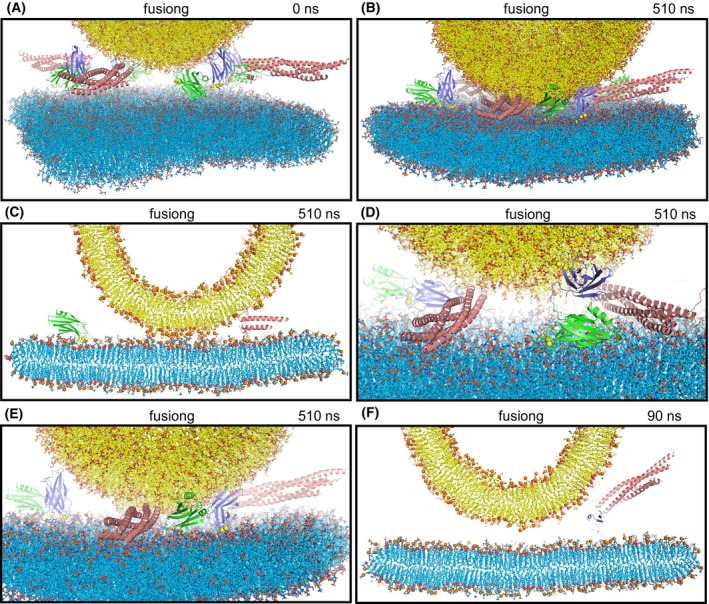
Molecular dynamics simulation designed to examine whether the Ca^2+^‐saturated Syt1 C_2_ domains cooperate with almost fully assembled SNARE complexes to induce membrane fusion (fusiong simulation). (A) Initial configuration. (B–E) Different views of the configuration after 510 ns of simulation showing the overall system (B), a slice of the center of the system to illustrate the contact between the vesicle and the flat bilayer (C), and close‐up views showing the SNARE complexes and C_2_AB molecules on one side of the system (D) or the other side (E). (F) Slice of the system after 90 ns of simulation showing how the membrane remained more distant than in an analogous simulation without Syt1 C_2_AB molecules (compare with Fig. 3C). Lipids are represented by stick models, proteins by ribbon diagrams and Ca^2+^ ions by spheres with the same color coding as in Fig. 1. In (C, F), phosphorous atoms of phospholipids and oxygen atoms of cholesterol molecules are shown as spheres.

**Fig. 3:**
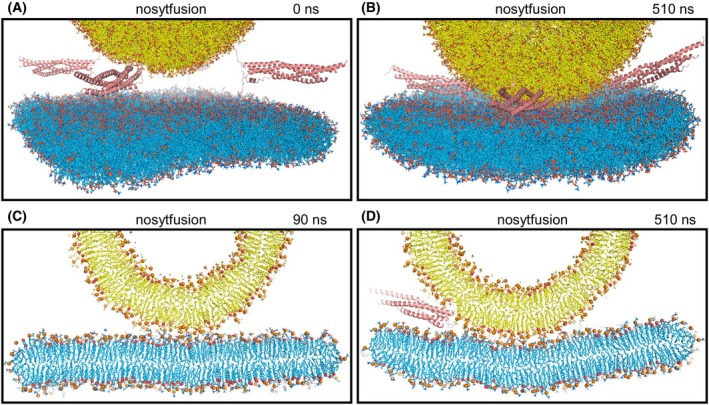
Molecular dynamics simulation of a system analogous to fusiong but without C_2_AB molecules to test whether they hinder the action of SNARE complexes in bringing membranes together (nosytfusion simulation). (A) Initial configuration. (B) Configuration after 510 ns of simulation. (C, D) Slices of the system after 90 ns (C) and 510 ns (D). Panel (C) shows how the membranes were already in contact at 90 ns, in contrast with the simulation that included C_2_AB molecules (Fig. 2F). Panel (D) shows that the two membranes started to form an extended interface, unlike the simulation including C_2_AB molecules (Fig. 2C). Lipids are represented by stick models, proteins by ribbon diagrams and Ca^2+^ ions by spheres with the same color coding as in Fig. 1. In (C, D), phosphorous atoms of phospholipids and oxygen atoms of cholesterol molecules are shown as spheres.

**Fig. 4:**
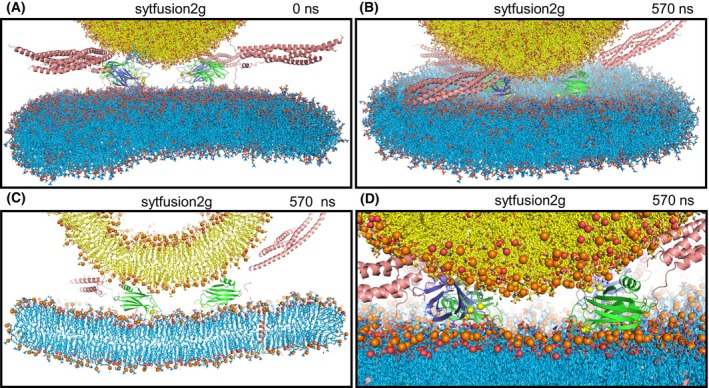
Molecular dynamics simulation with two Ca^2+^‐saturated Syt1 C_2_ domains located between the two membranes to study whether they might play a direct role in membrane fusion (sytfusion2g simulation). (A) Initial configuration. (B–D) Different views of the system configuration after 570 ns showing the overall system (B), a slice of the center of the system to illustrate the lack of contact between the vesicle and the flat bilayer (C), and a close‐up view that illustrates how the steric hindrance caused by the Syt1 C_2_ domains prevents the membranes from coming into contact (D). Lipids are represented by stick models, proteins by ribbon diagrams and Ca^2+^ ions by spheres with the same color coding as in Fig. 1. In (C, D), phosphorous atoms of phospholipids and oxygen atoms of cholesterol molecules are shown as spheres.

**Fig. 5:**
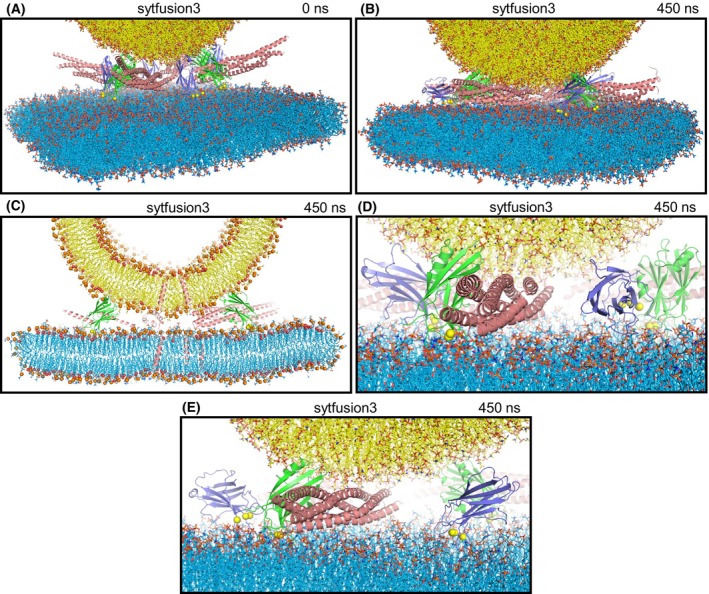
Molecular dynamics simulation designed to examine whether Ca^2+^‐saturated Syt1 C_2_ domains might act as wedges that prevent the membranes from coming closer while the SNARE complexes pull the membranes together in the center to induce torque forces that help to bend the membranes to initiate membrane fusion (sytfusion3 simulation). (A) Initial configuration. (B–E) Different views of the configuration after 450 ns of simulation showing the overall system (B), a slice of the center of the system to illustrate the lack of contact between the vesicle and the flat bilayer (C), and close‐up views showing the SNARE complexes and C_2_AB molecules on one side of the system (D) or the other side (E). Lipids are represented by stick models, proteins by ribbon diagrams and Ca^2+^ ions by spheres with the same color coding as in Fig. 1. In (C), phosphorous atoms of phospholipids and oxygen atoms of cholesterol molecules are shown as spheres.

For all systems, we used the same vesicle generated previously [24] and moved lipids manually to accommodate different positions of the SNARE TM regions. The cac2absc simulation used the same bilayer and SNARE complexes used for the prsg simulation of [28]. The four C_2_AB molecules were placed manually at positions interspersed between the four SNARE complexes, with the Ca^2+^‐binding loops pointing toward the flat bilayer but without being in contact with it (Fig. 1A). The fusiong system was analogous to the cac2absc system but using four copies of the SNARE complex that was almost fully assembled in fusiong and placing the C_2_AB molecules with the Ca^2+^‐binding loops pointing toward the center of the vesicle‐flat bilayer interface (Fig. 2A). The nosytfusion system had the same initial configuration of the fusiong system but without C_2_AB molecules (Fig. 3A). The sytfusion2g system also had a configuration analogous to fusiong but with only two C_2_AB molecules that had the Ca^2+^‐binding loops pointing in the same direction, toward the center of the vesicle‐flat bilayer interface (Fig. 4A). The sytfusion3 system included a vesicle, a flat bilayer and four trans‐SNARE complexes placed closer to the center of the bilayer‐bilayer interface, compared to the systems of Figs 1, 2, 3, 4, as in the fusion2g simulation of [15]. A restrained MD simulation was used to pull the syntaxin‐1 TM regions to designed positions such that the distance between the vesicle and the flat membrane was increased by 1.6 nm to allow sufficient space to place four C_2_AB molecules poised to bridge the two membranes (Fig. 5A).

Systems were energy minimized using double precision. The default mixed precision was used during production simulations as a compromise between simulation speed and accuracy. The systems were heated to 310 K running a 1 ns simulation in the NVT ensemble with 1 fs steps, and later equilibrated to 1 atm for 1 ns in the NPT ensemble with isotropic Parrinello‐Rahman pressure coupling [58] and 2 fs steps. NPT production MD simulations were performed for the times indicated in Table 1 using 2 fs steps, isotropic Parrinello‐Rahman pressure coupling and a 1.1 nm cutoff for non‐bonding interactions. Nose‐Hoover temperature coupling [59] used three different groups of atoms: (a) protein atoms; (b) lipid atoms; and (c) water and KCL ions. Periodic boundary conditions were imposed with Particle Mesh Ewald (PME) [60] summation for long‐range electrostatics.

## Results

All‐atom MD simulations have provided a powerful tool to visualize the neurotransmitter release machinery bridging two membranes [28, 61] and understand how the SNAREs mediate membrane fusion [15]. These simulations showed that neuronal SNARE complexes readily draw two lipid bilayers into contact even when the four‐helix bundles formed by the SNARE motifs of synaptobrevin, syntaxin‐1 and SNAP‐25 are not fully assembled at the C terminus. When the synaptobrevin and syntaxin‐1 helices were extended to the juxtamembrane (jxt) linkers that join the SNARE motifs with the transmembrane (TM) regions (referred to as jxt linker zippering), fusion between a flat bilayer and a vesicle bridged by the SNARE complexes occurred in < 2 μs at 350 K and was initiated because the jxt linkers and adjacent TM residues drawn into the polar bilayer‐bilayer interface catalyzed encounters of the hydrophobic lipid acyl chains at the interface [15]. To investigate how the Syt1 C_2_ domains might cooperate with the SNAREs in the events leading to membrane fusion and evaluate various models of Syt1 function that have been proposed, we performed all‐atom MD simulations of similar systems. All of them included four trans‐SNARE complexes bridging a vesicle and a flat bilayer with lipid compositions that resemble those of synaptic vesicles and synaptic plasma membranes, respectively [56, 57] (Table 1), with or without Syt1 fragments containing its two C_2_ domains (C_2_AB). Complexin was not included in the simulations for simplicity, as fast neurotransmitter release is impaired but not abolished in the absence of complexins [62] and we wanted to focus on how the functions of Syt1 and the SNAREs are coupled.

In a first simulation, we used a system of four trans‐SNARE complexes that were zippered to distinct extents at the C terminus and included four C_2_AB molecules bound to five Ca^2+^ ions each and with the Ca^2+^‐binding loops pointing toward the flat bilayer without contacting it (Fig. 1A) to study whether the Syt1 C_2_ domains spontaneously insert into the bilayers, perturb or bridge the bilayers, and perhaps even help initiate membrane fusion (referred to as cac2absc simulation). The SNARE complexes were in the same configurations and positions as those of a simulation of the primed state described previously (prsg simulation in Ref. [28]). During the 668 ns of this simulation, the two membranes were brought into contact (Fig. 1B,C), but there was no substantial progress in zippering of the SNARE complexes and the C_2_ domains of the distinct C_2_AB molecules exhibited different orientations with respect to each other, to the SNARE complexes and to the membranes (Fig. 1D,E). There were some interactions of the C_2_ domains with the SNAREs but they were all different from each other and some SNARE complexes did not bind to any C_2_ domain. One of the C_2_B domains had the Ca^2+^‐binding loops oriented toward the center of the membrane‐membrane interface and was bound to the C terminus of a SNARE complex that was almost fully assembled from the beginning of the simulation (Fig. 1F; same complex as that on the left side of Fig. 1D). This arrangement could facilitate cooperation of the SNAREs and the C_2_B domain in inducing membrane fusion, but it was already present early in the simulation (at 180 ns) and there was no initiation of fusion during the rest of the simulation.

We reasoned that the probability of observing membrane fusion in this simulation might have been hindered because three SNARE complexes were only partially assembled. Thus, we generated a similar system but with the four SNARE four‐helix bundles almost fully assembled and four C_2_AB molecules with the C_2_B domain Ca^2+^‐binding loops oriented toward the center of the membrane‐membrane interface (fusiong simulation) (Fig. 2A). We performed an MD simulation of this system for 510 ns and observed that the membranes came into contact, but we again did not observe initiation of fusion (Fig. 2B,C). As observed in the cac2absc simulation, the C_2_ domains of each Syt1 C_2_AB molecule adopted distinct orientations with respect to each other, with respect to the SNARE complexes and with respect to the membranes (Fig. 2D,E), and we did not observe any Syt1‐SNARE binding mode that resembled those of the cac2absc simulation.

To examine the possibility that the C_2_AB molecules might actually have hindered SNARE action in the fusiong simulation, we performed a parallel 510 ns simulation with an identical system that lacked the four C_2_AB molecules (nosytfusion simulation) (Fig. 3A). There was also no initiation of fusion in this simulation (Fig. 3B–D), but the two membranes came into contact more quickly than in the presence of C_2_AB molecules, as illustrated by comparing the slices of frames taken at 90 ns in the fusiong and nosytfusion simulations (Figs 2F and 3C, respectively). It is also noteworthy that at the end of the nosytfusion simulation the contact between the two bilayers was considerably more extensive (Fig. 3D) than at the end of the fusiong simulation (Fig. 2C). The extended interface formed during the nosytfusion simulation was observed in a previous simulation of a similar system [28] and in cryo‐electron microscopy (cryo‐EM) images of SNARE‐mediated liposome fusion reactions [50]. Hence, our results suggest that the C_2_AB molecules of the fusiong simulation hindered the action of the SNAREs in bringing the membranes together and the formation of extended interfaces, which also agrees with cryo‐EM data [63].

We also explored the notion that the Ca^2+^‐binding loops of both Syt1 C_2_ domains might play a direct role in membrane fusion if the C_2_ domains are located between the two membranes with the Ca^2+^‐binding loops oriented toward the center of the membrane‐membrane interface such that one of the loops can bind to the vesicle and the other to the flat bilayer. In this configuration, Ca^2+^‐binding might favor movement of lipids toward the Ca^2+^‐binding sites to destabilize the bilayers and initiate fusion. To test this idea, we performed a 570 ns MD simulation of a system analogous to the nosytfusion system but including two C_2_AB molecules between the membranes (sytfusion2g simulation) (Fig. 4A). We did not observe the postulated lipid movements during the simulation and the Syt1 C_2_ domains remained between the membranes throughout the simulation (Fig. 4B,C). A close‐up view of the last frame of the simulation illustrates the steric hindrance caused by the Syt1 C_2_ domains which, despite their small size, clearly prevent the membranes from coming into contact (Fig. 4D). These observations show that, when placed close to or at the site of fusion, the C_2_AB molecules hinder the action of the SNARE complexes in bringing the membranes together, which we also observed in MD simulations of SNARE complexes bridging two flat bilayers [28].

In the MD simulation with four trans‐SNARE complexes that had the jxt linkers zippered and yielded fusion of a vesicle and a flat bilayer, the SNARE complexes were closer to the center of the bilayer‐bilayer interface than in the simulations including Ca^2+^‐bound C_2_AB molecules described above, and this location likely facilitated the rapid induction of membrane fusion [15]. In this configuration, it is impossible for the Syt1 C_2_ domains to be in contact with the site of fusion because of steric hindrance. However, the C_2_ domains could be located further from the center, where they could bridge the two membranes as predicted in some models of Syt1 function [48, 49]. In such positions, the C_2_ domains might act as wedges that prevent the membranes from coming closer while the SNARE complexes pull the membranes together in the center, resulting in a torque that could help to bend the membranes to initiate fusion (as proposed previously for Munc18‐1 function [64]). To test this model, we started with a system with four trans‐SNARE complexes closer to the center that we generated previously (fusion2g in Ref. [15]). We increased the separation between the flat membrane and the vesicle by 1.6 nm to make room for the bridging C_2_ domains, used a restrained simulation to move the syntaxin‐1 TM regions to their positions in the translated flat bilayer, and included four C_2_AB molecules in positions in which the two C_2_ domains were poised to bridge the two membranes (Fig. 5A). We ran a 450 ns MD simulation (sytfusion3) and observed that the two membranes came closer to each other but were not brought into contact (Fig. 5B,C) because of steric hindrance caused by the C_2_ domains. Three of the four C_2_B domains and one of the C_2_A domains were bridging the vesicle and the flat bilayer at the end of the simulation (Fig. 5D,E), and hence could still be acting as wedges, but the SNARE complexes were unable to pull the bilayers together in the center.

**Fig. 6:**
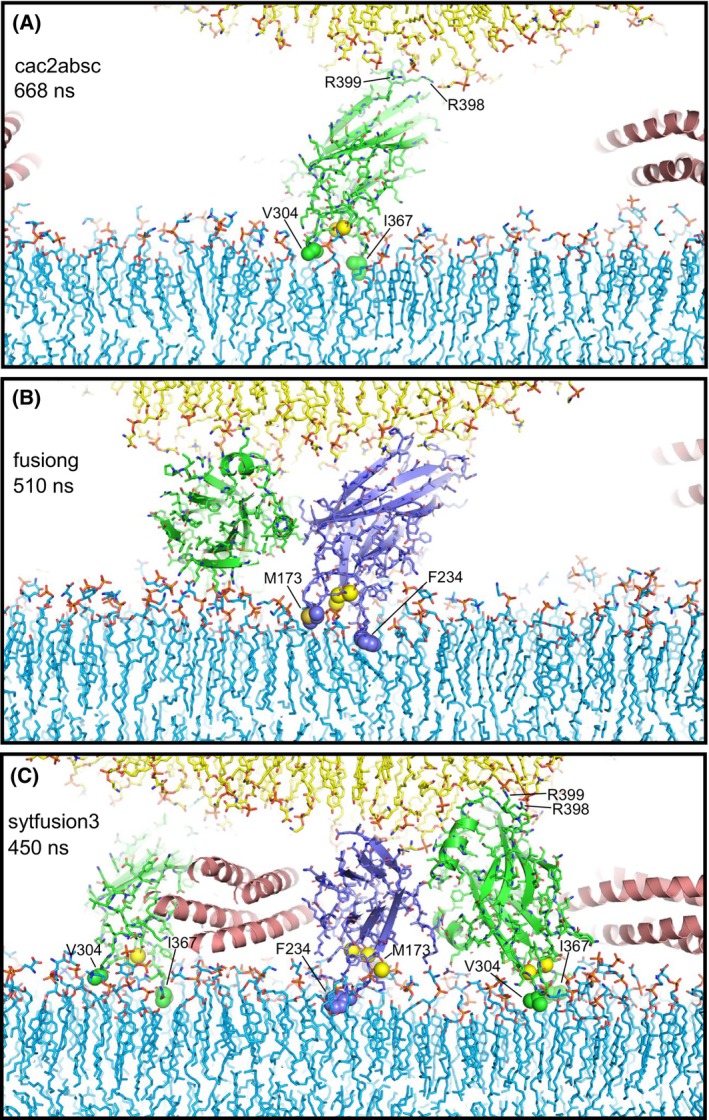
Syt1 C_2_ domain Ca^2+^‐binding loops do not insert deeply into lipid bilayers and cause limited bilayer perturbation during the simulations. The diagrams show examples of C_2_ domains with their Ca^2+^‐binding loops interacting with the flat bilayer from frames taken at 668 ns of the cac2absc simulation (A), 510 ns of the fusiong simulation (B) and 450 ns of the sytfusion3 simulation (C). Lipids are shown as stick models. Ca^2+^ ions are shown as yellow spheres. SNARE complexes are represented by ribbon diagrams and Syt1 C_2_ domains by ribbon diagrams and stick models with nitrogen atoms in dark blue, oxygen in red, sulfur in yellow orange and carbon colored in slate blue (C_2_A) and green (C_2_B). Other color coding is as in Fig. 1. The hydrophobic residues at the tips of the Ca^2+^‐binding loops that insert into the flat bilayer are shown as spheres and labeled. R398 and R399 at the opposite end of the C_2_B domain are labeled in (A, C) to illustrate how the C_2_B domain can bridge two membranes as predicted [48].

The results of all these simulations need to be interpreted with caution because of their limited duration. However, the overall results do suggest that C_2_ domains near the site of fusion hinder the action of SNARE complexes in bringing the membranes together. The simulations support the notion that the C_2_B domain can bridge two membranes through interactions of the Ca^2+^‐binding loops with one bilayer and binding of R398 and R399 at the other end of the β‐sandwich to the other bilayer [48, 49] (Fig. 6A). However, it is clear that such bridging does not cooperate with the SNAREs in bringing the two membranes together and in fact hinders this action by the SNAREs. Our simulations also support the notion that the Ca^2+^‐binding loops of both C_2_ domains can insert into membranes, with the hydrophobic residue at the tip of each loop contacting the hydrophobic acyl region of the flat bilayer [42, 43], as we observed such insertion very frequently in the simulations (Fig. 6A–C). However, the loops did not insert deeply into the bilayer, consistent with results from previous MD simulations [61]. Loop insertion caused local perturbations in the packing of the bilayer, but did not induce major alterations of the bilayer structure or substantial membrane curvature (Fig. 6). Hence, our simulations do not support the widespread models suggesting that Syt1 facilitates membrane fusion by perturbing bilayers, inducing membrane curvature or bridging membranes. While it seemed paradoxical that binding of the Syt1 C_2_B domain to the SNARE complex via the primary interface orients its Ca^2+^‐binding loops away from the fusion site [26], it now appears that this feature makes a lot of sense because it keeps Syt1 away from the fusion site, where it would hinder rather than facilitate SNARE action.

## Discussion

The role of Syt1 as the Ca^2+^ sensor that triggers fast, synchronous neurotransmitter release was clearly established two decades ago [29, 33] but, despite intense research, its mechanism of action has remained unclear. The crystal structure of the Syt1‐SNARE complex that revealed the primary interface [26] represented a seminal advance because it showed how the Ca^2+^ sensor and the fusion machinery are prepared to trigger fast membrane fusion in the primed state of synaptic vesicles. However, this structure also raised a conundrum, as the fact that Syt1 binding to the SNARE complex orients the C_2_B domain Ca^2+^‐binding loops away from the fusion site did not appear to be compatible with widespread models postulating that Syt1 helps the SNAREs trigger membrane fusion by perturbing bilayer packing [42, 44, 45], inducing membrane curvature [46, 47] or bridging the membranes [48, 49]. The MD simulations presented here help resolve this conundrum, casting further doubt on these models and suggesting that the Syt1 C_2_ domains hinder SNARE function if they are placed close to the site of fusion. These results suggest that, in addition to preparing the release machinery to trigger fast fusion upon Ca^2+^ influx, binding of Syt1 to the SNARE complex through the primary interface keeps Syt1 away from the fusion site such that it does not interfere with the actions of the SNAREs on the lipids. In fact, Syt1 may facilitate these actions of the SNAREs remotely by pulling the SNARE complex and facilitating jxt linker zippering as proposed in the lever model of Syt1 action [40, 41], which was developed in part because of the results of the MD simulations described here.

Our all‐atom MD simulations are limited by the simulation times that can be reached for multimillion atom systems even with the fastest supercomputers available, and therefore cannot completely rule out the widespread models of Syt1 function mentioned above. For instance, we did not observe strong bilayer perturbations or induction of substantial membrane curvature by the Syt1 C_2_ domains in the various simulations presented, all of which were ran for < 1 μs, but it is plausible that these events might occur at longer time scales and perhaps could be observable in simulations performed with higher temperatures, longer durations or sampling enhancing techniques. Nevertheless, the visualization of these systems and their behavior provided by the simulations can help enormously to assess the merits of mechanistic ideas that have been proposed for Syt1 action. Thus, while our simulations certainly support the notion that the single hydrophobic residue present at the tip of each Syt1 C_2_ domain Ca^2+^‐binding loop inserts into the hydrophobic acyl region of phospholipid bilayers [42, 43] (Fig. 6), it is also clear that such insertion can be readily accommodated in a bilayer without major perturbations because of the small size of the loops. There is no obvious reason why the resulting small perturbations should facilitate membrane fusion or should lead to induction of membrane curvature. There is also not overt reason why insertion of the Ca^2+^‐binding loops should cause larger perturbations than those observed in our simulations and others [61]. We note that fluorescent probes attached to cysteines at the tips of the Ca^2+^‐binding loops are expected to insert more deeply into the acyl region because they are larger than the native residues, which has led to the assumption that the Ca^2+^‐binding loops penetrate more deeply into the membrane (reviewed in Refs [64, 65]) than they actually do. Moreover, electron microscopy experiments supporting the notion that Syt1 induces membrane curvature used negative stain, which strongly perturbs membranes, and were performed with artificially high protein‐to‐lipid ratios (1 : 40) [46, 47] such that proteins should cover much of the lipid surface. In addition, previous simulations showed that SNARE complexes can readily bring membranes into contact without the help of Syt1 [15, 28] and the simulations including Syt1 C_2_AB presented here clearly show that this activity is hindered when the Syt1 C_2_ domains bridge the two membranes (Fig. 5). All these observations suggest that popular models postulating that Syt1 facilitates membrane fusion by bridging the membranes, perturbing the bilayers or inducing membrane curvature are incorrect.

**Fig. 7:**
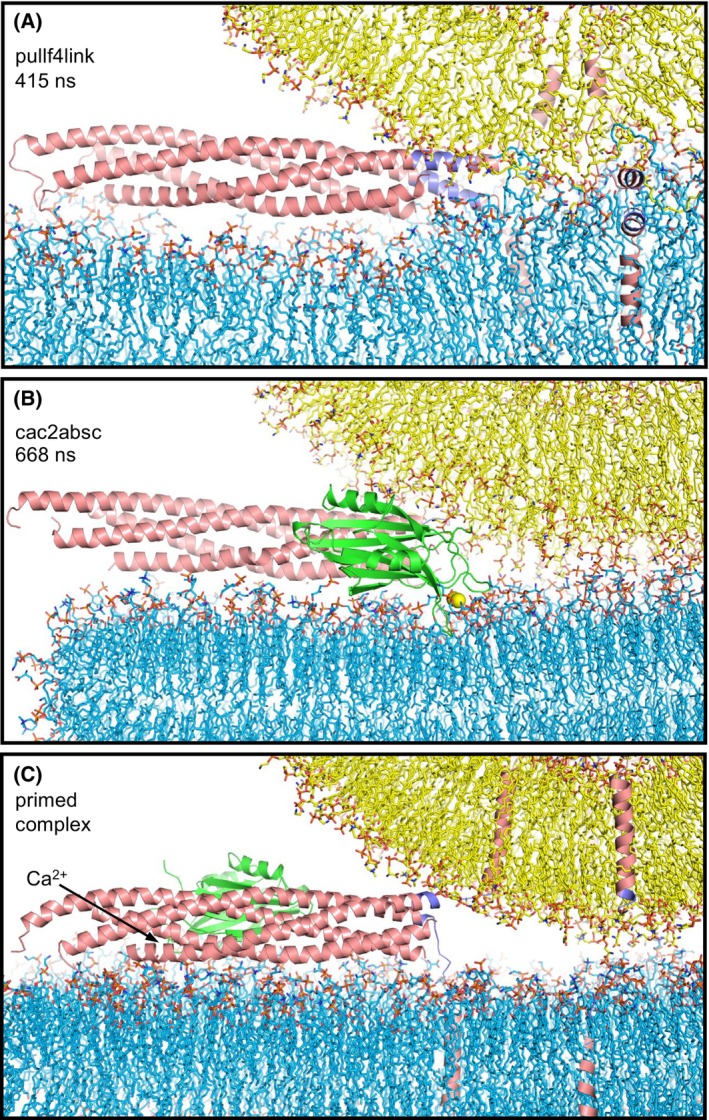
Visualization of key aspects of how the neurotransmitter release machinery is prepared to and may initiate membrane fusion. (A) Frame taken at 415 ns of a molecular dynamics (MD) simulation with the jxt linkers zippered (referred to as pullf4link) and catalyzing encounters between the acyl chains of the vesicle and the flat bilayers that initiated membrane fusion (described in Ref. [15]). (B) Close‐up view of the frame taken at 668 ns of the cac2absc simulation showing a C_2_B domain bound to the C terminus of a SNARE complex that is almost fully assembled and with the Ca^2+^‐binding loops oriented toward the center of the membrane‐membrane interface (same C_2_AB molecule showing in Fig. 1F but from a different angle and omitting the C_2_A domain for simplicity). (C) Model of the primed Syt1‐SNARE complex based on the crystal structure that revealed the primary interface [26] and MD simulations [28]. The C_2_A domain is omitted for simplicity. The arrow points to the C_2_B domain Ca^2+^‐binding loops. Lipids are represented by stick models, proteins by ribbon diagrams and Ca^2+^ ions by spheres with the same color coding as in Fig. 1 except that the jxt linkers of synaptobrevin and syntaxin‐1 are colored in slate blue.

This conclusion is further supported by the new model of SNARE‐mediated membrane fusion triggered by jxt linker zippering emerging from our previous MD simulations, which makes a lot of sense from a physicochemical point of view, is supported by extensive experimental evidence and does not postulate an important role of membrane curvature for initiation of fusion [15]. Instead, fusion is triggered because jxt linker zippering brings hydrophobic groups from the linkers and the TM region to the polar membrane‐membrane interface, which readily perturbs the bilayers without the need for Syt1 because the hydrophobic groups catalyze lipid acyl chain encounters at the interface (Fig. 7A). During all the MD simulations including Syt1 C_2_AB presented here, the only configuration that suggested a potential cooperation of Syt1 with the SNAREs in membrane fusion is that observed for one of the complexes in the cac2absc simulation, where the C_2_B domain is bound to the C terminus of the SNARE four‐helix bundle with the Ca^2+^‐binding loops projected toward the site of fusion (Fig. 1F). However, even in this configuration, it is difficult to imagine how the C_2_B Ca^2+^‐binding loops could cause a dramatic increase in the speed of fusion and, in fact, the C_2_B domain in this position could sterically hinder the action of the jxt linkers in bringing the membranes together at the site where they can catalyze the acyl chain encounters at the interface (compare Fig. 7B with 7A). Note also that, compared to the configuration of Fig. 7B, binding of the Syt1 C_2_B domain to the SNARE complex via the primary interface places C_2_B on the other side of the SNARE four‐helix bundle and with the Ca^2+^‐binding loops pointing in the completely opposite direction (Fig. 7C). Since it is now well established that Syt1 C_2_B domain binds to the SNARE complex via the primary interface in the primed state of synaptic vesicles [26, 41], reaching the configuration of Fig. 7B upon Ca^2+^ influx would require dissociation of the C_2_B domain from the SNAREs and dramatic rotations that do not bode well for the high speed of synchronous release, without an obvious driving force. Moreover, Ca^2+^ binding does not cause full dissociation of the Syt1 C_2_B domain from the SNARE complex, but rather partial dissociation driven by reorientation of the C_2_B domain on the membrane [40].

The MD simulations presented here led us to the realization that it was crucial to find alternative models of Syt1 function that involved a remote action rather than direct effects at the site of membrane fusion. This realization, together with evidence showing that jxt linker zippering is critical for neurotransmitter release [66, 67] and data showing that Ca^2+^ induces reorientation of the Syt1 C_2_B domain bound to membrane‐anchored SNARE complex to trigger neurotransmitter release, led us recently to develop the lever model of Syt1 action [40, 41]. In this model, Syt1 acts remotely from the site of fusion upon Ca^2+^ binding by pulling the SNARE complex and thus facilitating jxt linker zippering, which is hindered by the bent structure of the jxt linkers dictated by the natural geometry of the system and by interactions of the jxt linker residues with the lipids (illustrated in Fig. 7C). The notion that neurotransmitter release is triggered by Ca^2+^‐induced reorientation of the Syt1 C_2_B domain with respect to the SNARE complex is strongly supported by multiple correlations between the effects of mutations on binding of C_2_B to the SNARE complex via the primary interface and their effects on neurotransmitter release [40, 41]. However, multiple aspects of this model remain to be tested, including the potential effects that complexin can have in preventing fusion before Ca^2+^ influx and perhaps facilitating fusion when Ca^2+^ binds to Syt1. MD simulations of the primed state including complexin have already yielded important insights [28], supporting the notion that steric clashes of complexin with the vesicle hinder membrane fusion [68], and further simulations including complexin may help understand whether it can play an active role in triggering fusion upon Ca^2+^ influx.

The primary interface is conserved only in Syt isoforms that are closely related to Syt1 but not in other Syt isoforms or C_2_ domain proteins involved in membrane traffic [26]. Hence, while Syts and other C_2_ domain proteins have been widely believed to function by analogous mechanisms as Syt1, the majority of them do not function through a primary interface. Nevertheless, the key observations from the MD simulations presented here do not involve the primary interface but rather general features that are common to most Ca^2+^‐dependent C_2_ domains including those of Syt1 and hence have general implications to understand the mechanisms of action of the variety of C_2_ domain proteins that are involved in membrane traffic [51, 52]. Thus, the notion that C_2_ domains involved in membrane traffic act remotely rather than disrupting lipid bilayers, inducing membrane curvature, or bridging membranes, may be universal. Clearly, testing this prediction will require extensive further experimentation. The study described here emphasizes the power of MD simulations to illuminate complex molecular mechanisms and help to generate testable hypotheses.

## Conflict of interest

The authors declare no conflict of interest.

### Peer review

The peer review history for this article is available at https://www.webofscience.com/api/gateway/wos/peer‐review/10.1002/2211‐5463.13966.

## Author contributions

JR performed the study, and conceptualized and interpreted the simulations together with KJ and CR. JR wrote the manuscript, and KJ and CR revised the manuscript.

## Data Availability

Most files corresponding to the molecular dynamics simulations have been deposited in the dryad database (https://doi.org/10.5061/dryad.mgqnk9978). Because of the very large size of trajectory files, it was not practical to deposit them in this database, but these files are available from the corresponding author upon reasonable request.
